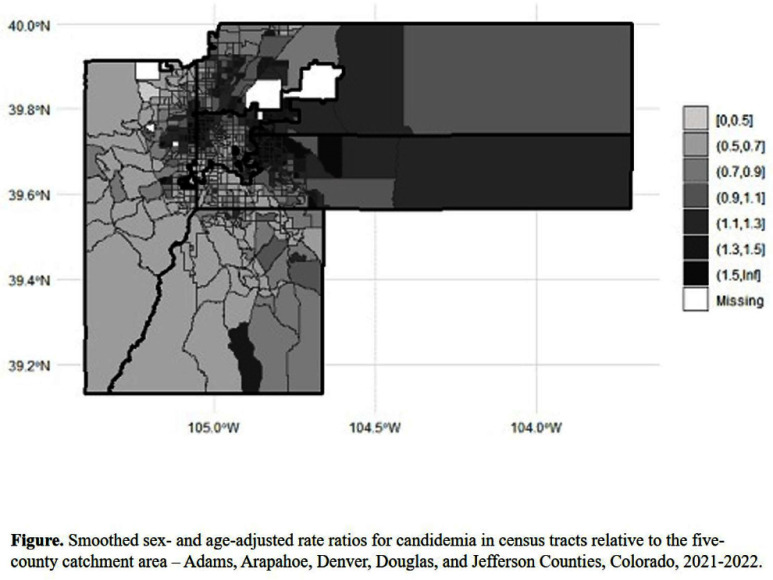# Candidemia Rates and Census Tract Social Vulnerability Index – Colorado, 2021-2022

**DOI:** 10.1017/ash.2025.382

**Published:** 2025-09-24

**Authors:** Christopher Czaja, Alyssa Beck, Kelsey Orten, Alison Abraham, Rebecca Conway, Johnna Bakalar, Helen Johnston

**Affiliations:** 1 Colorado Department of Public Health and Environment; 2 University of Colorado, Anschutz Medical Campus

## Abstract

**Background:** The association between social determinants of health (SDOH) and healthcare-associated infections is underappreciated. We evaluated disparities in rates of candidemia associated with the social vulnerability index in Denver area census tracts. **Methods:** We conducted an ecologic study of the 664 census tracts within Adams, Arapahoe, Denver, Douglas, and Jefferson Counties, Colorado in 2021 and 2022. The outcome of interest was census tract rates of candidemia, with cases captured by population-based surveillance of the Centers for Disease Control and Prevention’s (CDC) Emerging Infections Program and geocoded to census tract of residence. The primary explanatory variable was the CDC and Agency for Toxic Substances and Disease Registry Social Vulnerability Index (SVI) from 2022 and its four themes: socioeconomic status, household characteristics, race and ethnic minority status, and housing type and transportation. Population denominators were taken from the 2018-2022 American Community Survey, and census tract boundaries from the 2020 US Census. We used bayesian conditional autoregressive models to map smoothed rate ratios (RRs) of candidemia relative to the five-county catchment area rate and to estimate RRs and 95% credible intervals (CIs) associated with a 0.1 unit increase in SVI and each SVI theme. Analyses were conducted using SAS 9.4, RStudio 2023.06.0, and OpenBUGS 3.2.3 software. **Results:** Of 664 census tracts, 658 (99%) had a greater than zero population for which rates could be calculated. There were 333 candidemia cases in 5,652,254 person-years, of which 321 (96%) were geocoded and included in the analysis. Disease mapping indicated clusters of census tracts with higher than expected rates of candidemia relative to the five-county catchment area after adjusting for sex and age (Figure). For every 0.1 unit increase in census tract SVI, there was a 15% increase in the rate of candidemia (RR 1.15, 95% CI, 1.10, 1.20) after adjusting for sex and age. There were similar, modest associations with each SVI theme. **Discussion:** We demonstrated clustering of census tracts with higher than expected rates of candidemia and a modest association with SVI. Healthcare and public health epidemiologists should consider the contribution of SDOH to population rates of candidemia with an eye toward identifying opportunities to improve health equity.

**Figure:**